# Does Mutational Robustness Inhibit Extinction by Lethal Mutagenesis in Viral Populations?

**DOI:** 10.1371/journal.pcbi.1000811

**Published:** 2010-06-10

**Authors:** Eamon B. O'Dea, Thomas E. Keller, Claus O. Wilke

**Affiliations:** 1 Section of Integrative Biology, The University of Texas at Austin, Austin, Texas, United States of America; 2 Center for Computational Biology and Bioinformatics and Institute for Cellular and Molecular Biology, The University of Texas at Austin, Austin, Texas, United States of America; University of Western Ontario, Canada

## Abstract

Lethal mutagenesis is a promising new antiviral therapy that kills a virus by raising its mutation rate. One potential shortcoming of lethal mutagenesis is that viruses may resist the treatment by evolving genomes with increased robustness to mutations. Here, we investigate to what extent mutational robustness can inhibit extinction by lethal mutagenesis in viruses, using both simple toy models and more biophysically realistic models based on RNA secondary-structure folding. We show that although the evolution of greater robustness may be promoted by increasing the mutation rate of a viral population, such evolution is unlikely to greatly increase the mutation rate required for certain extinction. Using an analytic multi-type branching process model, we investigate whether the evolution of robustness can be relevant on the time scales on which extinction takes place. We find that the evolution of robustness matters only when initial viral population sizes are small and deleterious mutation rates are only slightly above the level at which extinction can occur. The stochastic calculations are in good agreement with simulations of self-replicating RNA sequences that have to fold into a specific secondary structure to reproduce. We conclude that the evolution of mutational robustness is in most cases unlikely to prevent the extinction of viruses by lethal mutagenesis.

## Author Summary

The high mutation rate of RNA viruses, such as HIV, allows them to rapidly evolve resistance to host defenses and antiviral drugs. A new approach to treating these viruses—lethal mutagenesis—turns the mutation rate of these viruses against them. It uses mutagens to increase the viruses' mutation rates so much that the accumulation of harmful mutations drives viral populations to extinction. Is there any way that a virus could adapt to a drug that increases its mutation rate? One way is that the virus could evolve so that mutations tend to be less harmful. In previous experimental work, there have been reports that virus populations can differ in robustness. Yet, the evolution of mutational robustness did not seem to inhibit extinction by lethal mutagenesis. In this work, we model viral populations under lethal mutagenesis in order to see when viruses might escape extinction by evolving robustness to mutations. We find that viruses can benefit from robustness only at relatively low mutation rates because the extent to which robustness increases fitness is rapidly drowned out by the extent to which higher mutation rates decrease fitness. The implication is that the evolution of mutational robustness is not a fundamental impediment to lethal mutagenesis therapy.

## Introduction

Lethal mutagenesis is a proposed therapy for patients with viral infections. The general approach is to increase the deleterious viral mutation rate enough so that the viral population will go extinct [1]. Here, we analyze the risk that lethal mutagenesis therapy will fail as a result of the virus population evolving mutational robustness.

Research on lethal mutagenesis and the question of how much mutational robustness can affect mutagenesis are of practical importance. In support of the promise of lethal mutagenesis as a treatment for many human and agricultural viruses, there are reports of the addition of a mutagen severely reducing or extinguishing populations of coxsackievirus B3 [2], foot-and-mouth disease virus [3]–[6], Hantaan virus [7], [8], hepatitus C virus [9], human immunodeficiency virus type 1 (HIV-1) [10], lymphocytic choriomeningitis virus (LCMV) [11]–[14], poliovirus [2], [15], [16], and vesicular stomatitis virus (VSV) [15], [17]. Several recent works have started to develop a theoretical framework to describe lethal mutagenesis [18]–[22]. Theoretical work has led to the prediction that lethal mutagenesis could also be a viable treatment for bacterial infections [20], [22].

An important limitation to any pathogen treatment is the ability of the pathogen to develop resistance. Since lethal mutagenesis introduces deleterious mutations throughout the genome of viruses, it seems that there are only two types of effective resistance mechanisms. First, the virus could evolve a mechanism to reduce the number of mutations that the therapeutic mutagen introduces. Ref. [23] described such resistant mutations in poliovirus being treated with ribavirin and Ref. [24] described them for foot-and-mouth disease virus. Second, the virus could evolve so that the mutations introduced become, on average, less deleterious. In other words, it could evolve to have greater sequence neutrality or mutational robustness.

Empirical studies of lethal mutagenesis appear to yield conflicting results. While Ref. [25] has provided evidence that two strains of VSV differed in mutational robustness during mutagenesis treatment, Ref. [14] later concluded from work with LCMV that lethal mutagenesis does not lead to the evolution of greater mutational robustness. Here, we explain how these apparently contradictory results are both consistent with a simple model of lethal mutagenesis.

The organization of this paper parallels our line of inquiry. First we ask, when will a population at equilibrium go extinct? We find with a deterministic model that an approximation for the critical mutation rate, i.e. the mutation rate beyond which the population goes extinct, is the log of reproductive capacity divided by the non-neutrality of the population at equilibrium. The implication is that small increase in the mutation rate can compensate for relatively large increases in neutrality. Next, we ask, how will elevating the mutation rate increase the rate at which populations move to areas of a neutral network with higher equilibrium neutrality? We find with a semi-deterministic model that the time it takes for a population undergoing mutagenesis to find the optimal area of the network grows exponentially with the size of the barrier to it. The implication is that we can usually disregard these shifts of the virus population, since the population will quickly shift to the optimal area if the barrier is small and the population will stay where it begins if the barrier is large. Finally, we ask, when will a population that is not at equilibrium go extinct? We show with a stochastic analytical model and simulations based on RNA-secondary structure networks both the critical mutation rate in these more complex models and the probability of stochastic extinction at mutation rates below the critical mutation rate. The implication is that the initial robustness of the population can be important in some cases, but not when the mutation rate exceeds the critical mutation rate.

## Results

### Deterministic theory

First, we consider the effects of mutational robustness in a deterministic model of lethal mutagenesis. In general, virus extinction is guaranteed if [18]

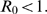
(1)

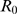
 is the basic reproductive ratio known from epidemiology. In the context of lethal mutagenesis, it measures the mean number of offspring virions (per infecting virion) that successfully infect a susceptible cell. 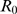 combines the effects of both virus reproduction and virus death. Offspring virions that die before having the chance to infect a susceptible cell do not contribute to 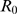.

We can write 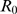 as 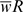 [18]. 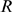 is the basic reproductive capacity of the best genotype in the viral fitness landscape and 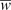 is the mean fitness of the viral population, measured in units of 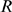. We use the term reproductive capacity for 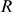 since no individual of any genotype can have a greater expected number of reproductive offspring. We assume that changes in the mutation rate affect only 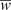 and leave 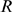 unchanged. Under the fairly weak assumptions that populations are large, recombination is absent, and mutations are Poisson-distributed [18], we have 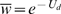. Thus, we can rewrite Equation (1) as
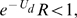
(2)where 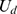 is the deleterious genomic mutation rate. Equation (2) allows us to solve for the deleterious mutation rate beyond which extinction is guaranteed. We find that 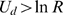 leads to extinction.

In general, we can write the deleterious mutation rate as 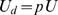, where 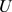 is the overall genomic mutation rate and 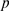 is the probability that a random mutation is deleterious. Equation (2) then becomes
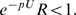
(3)Mutagenesis will increase 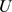. The evolution of mutational robustness will decrease 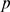.

Throughout the remainder of this paper, we consider populations evolving on neutral networks. All sequences on the neutral network have the same reproductive capacity 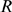, and sequences off the neutral network are inviable. The neutral-network metaphor is a reasonable approximation for populations near the top of their fitness peak in more general fitness landscapes. Strongly deleterious mutations will generally be purged from the population quickly and thus can be considered lethal. Weakly deleterious mutations will have a minor effect on population fitness and can—to first order—be considered as neutral mutations.

In the case where neutral sequences are distributed at equal density throughout the mutational network, 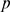 is a constant and corresponds to the fraction of non-neutral mutational neighbors at each node in the network. More generally, 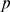 is determined approximately by the average population neutrality at equilibrium. This approximation has lead to good predictions for fitness landscapes based on RNA secondary-structure folding [26]. To first order, 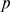 is independent of the mutation rate, because the average neutrality of a population depends primarily on the structure of the neutral network [27], [28]. However, for very large mutation rates, 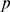 will depend on 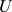 [29]. For example, for 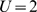, the number of a sequence's neutral two-point mutants will have a larger effect on the average neutrality than the number of neutral one-point mutants.

**Figure 1:**
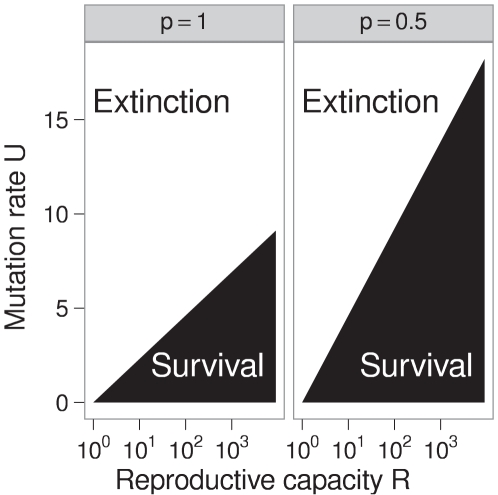
Effect of robustness on virus population survival. The set of mutation rates and reproductive capacities that allow the virus population to survive according to Equation (3) are shaded. This set is smaller in the absence of mutational robustness (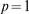) than in the presence of considerable robustness (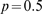), but the relationship between 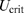 and 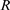 is consistently log-linear.

Under the assumption that 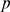 is independent of 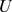, we can rearrange Equation (3) and solve for the value of 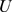 that must be exceeded for the population size to deterministically decrease. Throughout this paper, we denote this value of 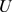 as 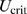 and for this deterministic model we find that
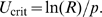
(4)As long as the critical mutation rate is close to unity and we use a 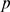 value measured at equilibrium, this expression will give a reasonable approximation for the critical mutation rate. Figure 1 shows how an increase in mutational robustness, i.e., a decrease in 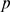, extends the regime in which a viral population can survive mutagenesis treatment.

Of course, the critical mutation rate may be far above unity and the assumption that 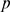 is independent of 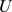 may not be valid in that regime. The stochastic models we analyze below indicate a way to make an analogous measurement in this case for the purpose of calculating 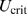. Before presenting that result, however, we next consider a more troubling possibility: Will the elevation of the mutation rate during lethal mutagenesis increase the rate at which the virus population evolves to a higher equilibrium level of robustness?

### Lethal mutagenesis in the neutral-staircase landscape

In general, a neutral network may be broken into separate areas of differing neutrality and separated by entropic barriers. (The term *entropic barrier* means that the probability to jump from one network to another with one mutational event is low.) In other words, there may be few possible paths in the network from one area to another. In this case, there is the risk that increasing mutation rates will increase the rate at which virus populations find rare paths to other areas of the neutral network in which it is possible to evolve greater neutrality. This process is comparable to that of demes drifting between equilibria (adaptive peaks) in the context of shifting-balance theory [30].

Depending on how great a barrier is in comparison to the mutation rate, the evolution of greater neutrality during lethal mutagenesis will be either inevitable or extremely unlikely. The barriers between areas of the neutral network at high mutation rates will often be so small that they can be neglected. In this case, the separate areas form one large, connected neutral network. Alternatively, the barriers will be so large that we may disregard the undiscovered areas of the neutral network. We next illustrate this concept with a specific example.

We consider the *neutral-staircase* landscape [29], a fitness landscape consisting of multiple nested neutral networks. Networks with relatively low connection density are embedded into larger networks with increasingly higher connection density. To discover the next larger network, a population has to cross an entropic barrier.

Sequences in the neutral-staircase landscape consist of zeros and ones (bits). The bits are organized into 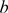 blocks of 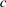 pairs of bits. Each block is separated by an additional 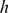 bits. The total sequence length is thus 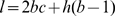. Blocks can be either *active* or *inactive*. Sequences are viable if and only if all bits in inactive blocks are set to zero and no pairs of bits in active blocks are both set to one. Viable sequences with minimal neutrality contain one active block at one end of the sequence and sequence neutrality increases when the inactive block adjacent to an active block becomes active. The inactive block adjacent to an active block becomes active when the 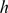 bits between the adjacent inactive and active blocks are all set to one at the same time. Thus, the 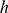 bits between blocks form an entropic barrier. The larger 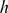, the harder it is to discover the more-densely connected areas of the neutral network.

The neutral-staircase landscape can be solved analytically, and the full derivation can be found in Ref. [29]. We express the solution in terms of the bit-copying–fidelity rate 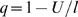 and the reduced mutation rate 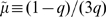. The average fitness of a population at equilibrium is given by
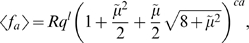
(5)under the assumption that the dominant sequence in the population has 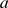 active blocks. To increase the number of active blocks, the population has first to generate a mutant with 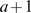 active blocks, and then this mutant has to go to fixation. The probability that at least one offspring sequence in one time step will have 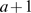 active blocks is
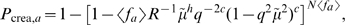
(6)where 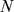 is the population size. A sequence with 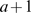 active blocks will become fixed with probability 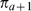. We obtain 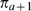 from the classic expression for the probability of fixation, 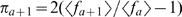 [31]. We can combine 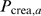 and 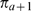 to estimate 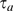, the expected number of generations until the dominant sequence changes from having 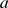 active blocks to having 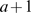 active blocks [29]:
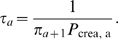
(7)(This expression assumes that the time to fixation is negligible compared to the time to discovery.)

If we sum 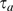 over all possible values of 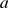, we obtain the convergence time, i.e., the expected time for the population to move from having one active block to the maximum number of active blocks, 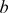:
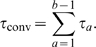
(8)


**Figure 2:**
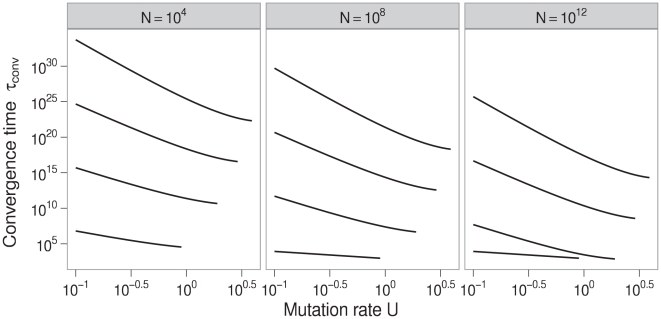
Expected time to evolve maximum robustness. In the neutral-staircase fitness landscape, the maximum neutrality increases as the number of active blocks increases. The expected time, in generations, for the number of active blocks 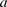 to go from one to a maximum number 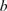 of 20 is plotted using Equation (8). The curves in each panel, from lowest to highest, correspond to the number of between-block bits 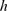 being 2, 4, 6, and 8. The curves for the lowest barrier can be fairly flat because fixation probabilities become the rate-limiting factors. Parameters: number of bitpairs per block 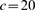, reproductive capacity 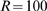.

Figure 2 shows convergence times as a function of mutation rate. The curves in Figure 2 are only plotted for 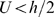, where Equation (8) has previously been found to be in good agreement with simulations [29]. When barriers are large, there is a log-log relationship between convergence time 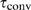 and the genomic mutation rate 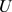. So convergence times may decline quickly as the mutation rate increases. However, there is a log-linear relationship between the convergence time and the size of the barrier. Therefore, even at high mutation rates, the time to convergence may be an astronomical number of generations if the barrier is large (Figure 2). This is true even for large populations.

**Figure 3:**
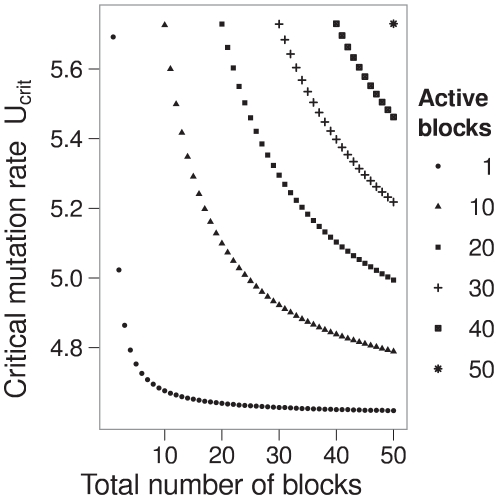
Critical mutation rates in the neutral-staircase fitness landscape. Critical mutations rates derived from Equation (5) are plotted as a function of the total number of blocks 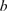 for varying numbers of active blocks 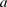. The critical mutation rates increase slowly as the number of active blocks increases. Parameters: number of bitpairs per block 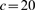, reproductive capacity 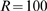, between-block bitstring length 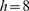.

The prospect of the equilibrium neutrality increasing raises the question of how much increases in equilibrium neutrality may increase 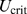. Although the calculation of convergence times assumed that that the population size was constant, we can answer this question by considering Equation (5) as a measure of absolute fitness. Then we find that an increase in the number of active blocks does not greatly increase the critical mutation rate 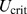 (Figure 3).

When barriers are small, we can expect that the area of the neutral network with the greatest connection density can be found in a reasonable number of generations. In this case, the main question is whether the population can find areas with high connection density before it goes extinct under mutagenesis. In the following subsections, we will address this question using fully stochastic models.

### Stochastic theory

According to Equation (1), extinction is guaranteed if the mutation rate is so high that the equilibrium mean fitness of the population is less than 1. But lethal mutagenesis is not an equilibrium process. Therefore, we next explore how extinction occurs in a population out of equilibrium, using the mathematical framework of multi-type branching processes. Because this approach is a stochastic one, we calculate not only the mutation rate at which extinction is guaranteed but more generally the probability that extinction happens at any given mutation rate. Our main question here is how the extinction probability changes if the population resides initially in regions of the neutral network with particularly low or high connection density.

The mathematical framework we use to calculate the extinction probability under lethal mutagenesis is that of multi-type branching processes. This framework has been used previously to calculate the fixation probability of a rapidly mutating virus on a neutral network [32], [33]. The next two paragraphs offer a brief introduction.

Consider a population where all offspring are identical to their parents. A sequence produces a random number of offspring in the next generation. All these offspring sequences produce their own random number of offspring according to the same probability distribution. The number of progeny that a sequence has in two generations, then, is the sum of these random variables. The use of a probability generating function (p.g.f.) allows for convenient expression of these sums. We use
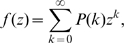
(9)where 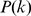 is the probability that the number of offspring equals 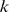. The convenience of using p.g.f.s is that we obtain the p.g.f. for the distribution of sizes for the second, third, and all following generations by iteratively substituting the p.g.f. 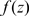 into itself two, three, or more times. The theory of branching processes [34] shows that the probability 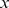 of extinction, the condition in which all sequences stop producing offspring, is the value of 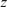 that satisfies the simple expression
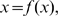
(10)so long as the expected number of offspring 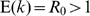 but finite. The theory also shows that the condition 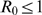 guarantees extinction.

When there is a finite number 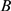 of distinct genotypes, we use multivariate offspring distributions. In this case, the p.g.f. is a vector-valued function and takes a vector 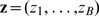 as its argument. Component 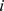 of the p.g.f. 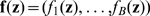 has the form
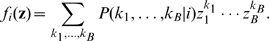
(11)Here, 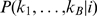 is the joint probability that genotype 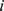 has 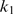 offspring of type 1, 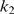 offspring of type 2, and so on. As in the one-dimensional case, the extinction probability follows from the fixed-point equation
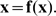
(12)Component 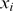 of the fixed point 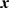 gives the probability that the branching process goes extinct if it was started with a single particle of type 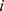, as long as the following assumptions are met [34]: The expectation and variance of the offspring of each type are finite; all types do not have exactly one offspring; each type can have a descendant of any other type; and the dominant eigenvalue 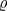 of the matrix of means is greater than one. The matrix of means, here denoted 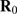, in a multi-type branching process is comparable to the expected number of offspring 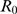 in a single-type branching process and has elements
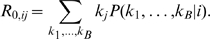
(13)If the above assumptions are satisfied except that 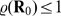, extinction is guaranteed.

Extinction probabilities can easily be found numerically from Equation (12), but we next present two approximations to illuminate how extinction probabilities follow from offspring distributions.

First, we need an explicit expression for the multivariate p.g.f.s in the fixed-point equation. If the number of offspring of type 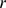 produced by a type-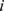 sequence is Poisson-distributed with mean 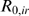, then Equation (9) defines the corresponding p.g.f. as 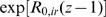. The p.g.f. for a sum of independent random variables is the product of the p.g.f.s of all the variables. Assuming independence of the number of offspring of each type, then, our multivariate p.g.f.s are
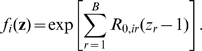
(14)


When extinction probabilities 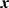 are close to one, we can approximate them by taking the log on both sides of Equation (12), expanding 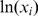 to second order, and performing some algebra to obtain
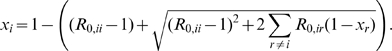
(15)Equation (15) says that the probability of extinction of a type-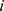 sequence is approximately 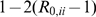 if this sequence does not produce any other types of sequences. This is natural since 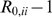 is a measure of how much the replication rate of type-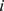 sequences exceeds the replacement rate. If we equate 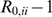 with the selective advantage 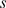 in a constant–population-size model, we see the classic result 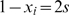 [31]. We also see in Equation (15) how the probabilities 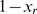 that other types of sequences do not go extinct weight the contribution of the rates 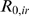 in reducing the extinction probability.

When extinction probabilities 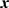 are close to zero, we can express 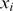 using the linear approximation of Equation (14) at zero:
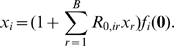
(16)Equation (16) says that 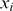 is at least the probability 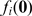 that a type-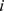 sequence produces no offspring. The equation also shows how 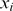 further increases as the fraction of offspring that will go extinct, 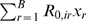, increases. Solving Equation (16) gives
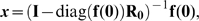
(17)where 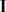 is the identity matrix and 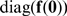 is the diagonal matrix whose diagonal elements are the elements of the vector 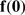.

### Stochastic extinction on an RNA secondary-structure network

The previous subsection developed the general theory of stochastic extinction under lethal mutagenesis. We will now apply this theory to the special case of a neutral network of RNA sequences. To this end, we will first describe a model that links a sequence's location in a neutral network with the sequence's neutrality. This model yields the rates 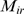 at which sequences produce offspring sequences with different levels of neutrality. We then present both analytic and simulation results that show how the initial location of a population affects its extinction probability.

Consider how the probability-density function of the offspring distribution 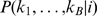, the probability that a sequence will produce any number of offspring with any combination of neutralities, depends on a sequence's location in a neutral network. The sequence's location determines how many mutations can push sequences off of the neutral network. The sequence's location also determines how mutations can change the fraction of a sequence's neighbors that are neutral (i.e. change the sequence's neutrality or robustness). In theory, we could determine the graph that connects all sequences in a neutral network, and read off 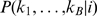 from this graph. But in practice, this graph is so large for RNA sequences of even modest length that this approach is not feasible. A more feasible, but still computationally intensive, approach would be to group sequences into classes of various levels of neutrality and then estimate a matrix of means from a sample of sequences from each class. The principle eigenvalue of this matrix of means would indicate if extinction was guaranteed. Instead, we here describe a sequence simply by two parameters 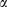 and 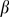. The parameter 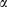 measures the probability that mutant offspring are neutral, and the parameter 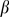 determines whether this probability stays constant (no epistasis), increases (antagonistic epistasis), or decreases (synergistic epistasis) as the number of mutations increases.

We define 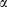 such that the larger it is, the *smaller* the probability that offspring are neutral (see next paragraph). Instead of 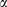, we also use the fraction of deleterious mutations 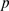, which satisfies
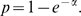
(18)The larger 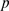, the smaller the probability that offspring are neutral. As in the deterministic model, 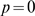 means that all offspring are neutral and 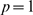 means that no offspring is neutral.

Our approach is inspired by Ref. [35], which showed that the fraction of neutral sequences at a distance 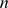 from a reference sequence decays approximately as
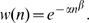
(19)Ref. [35] also showed that 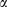 and 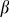 are not independent from each other, but that either parameter determines the other. The relationship between 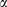 and 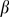 arises because the total number of neutral sequences in a given neutral network is a constant, 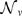. We can express 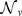 in terms of 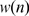 as
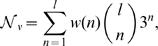
(20)where 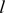 is the sequence length and 3 represents the number of RNA bases to which an existing base can mutate. Using Equation (19) for 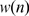 and given either 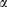 or 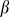, we can solve Equation (20) for the other parameter.

Equations (19) and (20) say that, since there are only so many neutral sequences, if a sequence is in an area of the neutral network with a high connection density, then the connection density of neutral sequences must generally decline as we move away from it, and vice versa. This reasoning implies that 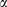 and 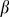 are negatively correlated, and we found here that 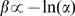 (Figure S1).

We can use this framework to determine the 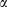 and 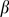 of an offspring sequence, given that we know 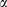 and 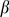 of the parent sequence. Equation (19) describes the expected density of neutral sequences as we move away from the parent sequence. The fraction 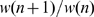 is the factor by which the probability of an offspring being neutral is reduced as the number of mutations goes from 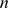 to 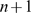. We take this fraction as the neutrality 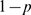 of an offspring with 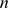 mutations. Then, 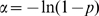. Note that this approach neglects back mutations, which generally are highly unlikely for sufficiently long sequences. Once we have the offspring's 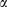, we can solve for the offspring's 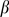 using Equations (19) and (20). We close this system by evenly dividing the range of the continuous variable 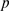 into 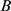 bins. Sequences with a 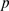 in the range of a bin are given the 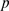 value of the upper boundary of the bin. The bins are indexed so that the 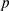 of type-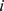 sequences 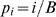.

Putting everything together, the probability that any one offspring of a parent of type 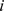 is of type 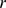 is
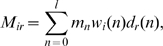
(21)where 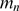 is the probability of having 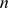 mutations, and 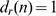 if 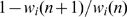 is in 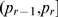 and 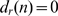 otherwise. To fully specify 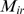, we assume that the distribution of mutations is Poisson with mean 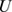. As explained in the previous subsection, if the number of offspring of each type are independent and Poisson-distributed, the p.g.f.s for the fixed-point equation used to calculate extinction probabilities are products of Poisson p.g.f.s. See Text S1 for a more detailed derivation.

**Figure 4:**
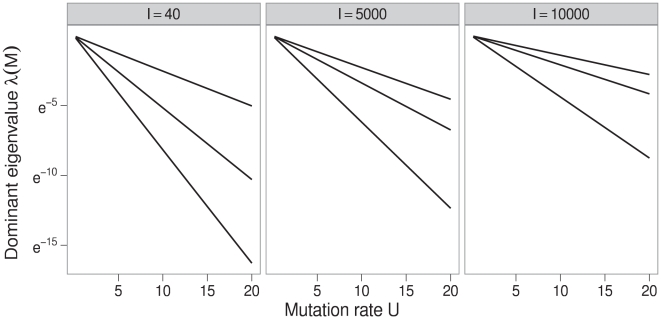
Effect of mutation rate on the dominant eigenvalue of the matrix 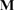. The dominant eigenvalue decays exponentially with the genomic mutation rate 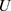 and the slope of the decay for a given sequence length is largely determined by the proportion of neutral sites in the sequences. The panels are labeled with the sequence lengths of 40, 5,000 and 10,000. For each sequence length, the lines, from lowest to highest, are numerical solutions where 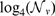 was set to approximately one third, two thirds, and five sixths of the sequence length. This gave neutral networks sizes equivalent to those from fitness landscapes in which approximately one third of sites are neutral, two thirds of sites are neutral, and the same proportion of sites are neutral as for the sequences in the RNA simulations in Figure 5.

The matrix 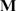 defined in Equation (21), multiplied with the reproductive capacity 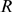, corresponds to the matrix of means 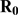 discussed in the previous subsection. Therefore, the critical mutation rate 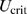 is the mutation rate at which the dominant eigenvalue of 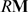 equals one. Here, 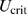 is determined by the parameters sequence length 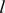, neutral-network size 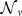, and reproductive capacity 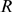 according to
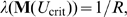
(22)where 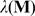 is the dominant eigenvalue of 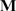 and represents the fraction of offspring produced at equilibrium that are neutral. 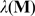 is an exponentially decaying function of 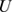 (Figure 4). Since 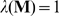 when 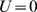, we can derive the rate of decay 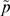 of 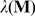 with 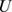 by measuring 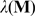 at a positive mutation rate 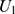:
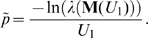
(23)This 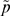 is an effective value of the probability of neutrality 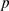 from the deterministic model subsection, and 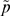 allows us to calculate critical mutation rates that are far above one as
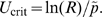
(24)

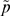
, and thus 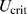, is largely determined by 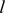 and 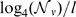 (Figure 4). The relationship between 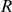 and 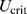 in Equation (24) is the same as in the deterministic model (Equation (4)). We next present results directly showing the relationships between 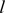, 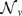, and 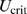.

**Figure 5:**
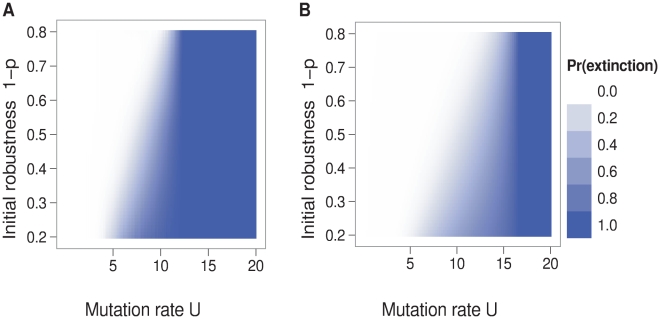
Extinction probability as a function of initial neutrality and genomic mutation rate. Panel A displays results from simulations where sequence neutrality was determined by RNA folding. Panel B displays results from a branching process model derived from the correlation between sequence neutrality and epistasis. Only in a band of intermediate mutation rates does the extinction probability depend on initial neutrality 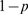. Parameters: sequence length 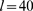, neutral-network size 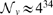, reproductive capacity 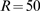, initial population size = 1 sequence.

First, we present results based on the assumption that populations initially consist of a single sequence. This case is relevant to a scenario in which a patient is inoculated with a small dose of virus while on lethal mutagenesis therapy or a virus is establishing itself in a new tissue of a patient's body. With this assumption, we found that the probability of extinction declined with the initial sequence's neutrality, but also that the gradient in extinction probabilities rapidly leveled as the mutation rate increased (Figure 5). In agreement with the theory of branching processes, the critical mutation rate 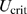 at which extinction is guaranteed was independent of the initial sequence's robustness.

**Figure 6:**
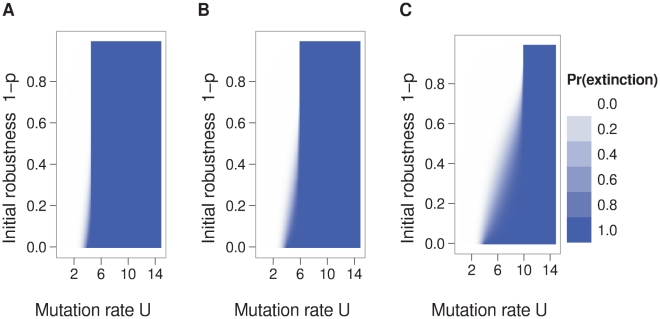
Effect of neutral-network size on extinction probability. The sizes of the neutral networks in panels A, B, and C are 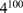, 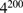, and 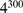, respectively. The dependence of the extinction probability on the initial robustness is greatest in panel C, where the neutral network is largest. These results are from a branching process model derived from the correlation between sequence neutrality and epistasis. Parameters: sequence length 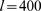, reproductive capacity 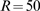, initial population size = 1 sequence.

Next we used the analytic calculations to study the effect of the size of the neutral network. When going from a smaller neutral network to a larger neutral network, the extinction threshold 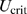 slowly moves towards larger values (Figure 6). Extinction probabilities decline faster with increasing 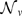 for populations that initially are highly robust (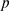 is small) compared to populations that initially are not very robust (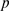 is large). Consequently, the larger the neutral network, the stronger is the extinction probability affected by the robustness of the sequence seeding the population (Figure 6).

**Figure 7:**
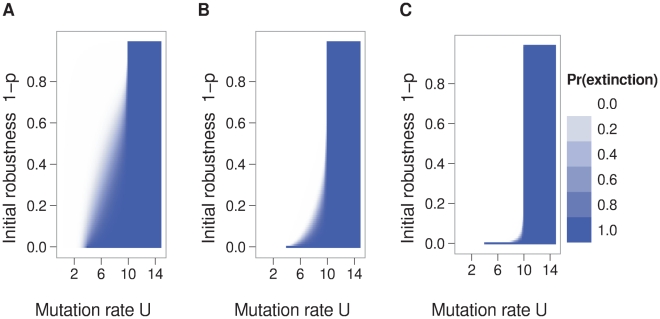
Effect of the initial population size on extinction probability. The initial population sizes in panels A, B, and C are 1, 100, and 100,000, respectively. The dependence of the extinction probability on the initial robustness is greatest in panel A, where the initial population size is small. These results are from a branching process model derived from the correlation between sequence neutrality and epistasis. Parameters: sequence length 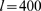, reproductive capacity 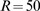, neutral-network size 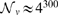.

Since lethal mutagenesis is intended to eliminate virus populations that have grown to high levels, we also considered the effect of the initial population size. We considered an initial population that was uniformly composed of sequences with a given initial robustness 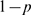. When going from a smaller initial population to a larger initial population, only the extinction probabilities for mutations rates below the extinction threshold changed (Figure 7). The gradient of extinction probabilities receded into a region in which sequence neutrality was low and mutation rates were just below the threshold. As in Figure 1A, the extinction threshold with 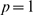 was the mutation rate where the expected number of offspring without any mutations was one, i.e. 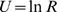. When the initial population was large and had at least a small amount 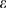 of neutrality (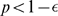), the extinction threshold was the mutation rate where, at equilibrium, the expected number of offspring without any mutations was one, i.e. 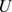 such that the eigenvalue of 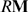 was one (Figure 7C).

We verified our branching-process model by carrying out simulations with individual RNA sequences (see Methods for details). The simulations used an RNA-folding algorithm to obtain a computationally tractable genotype-to-phenotype mapping that did not make the simplifying assumption that a sequence is fully described by just the two parameters 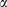 and 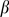. The simulations were initiated with sequences having a wide range of neutralities, as measured from the fraction of point mutations that maintained the neutral phenotype. In each generation of the simulations, sequences with the neutral phenotype reproduced, their offspring received a random set of mutations, and the phenotypes of these offspring were then determined. Simulations were continued until each population exploded or went extinct. The length of the sequences was 40. We found that the analytic calculations and the RNA secondary-structure simulation results were in broad agreement (Figures 5 and S2). The main difference was that the analytic calculations had a 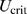 of roughly one to two mutations per replication above the 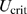 in the simulations.

## Discussion

We have studied how the evolution of mutational robustness affects lethal mutagenesis. Using a simple deterministic theory, we found that extinction was guaranteed past a critical mutation rate 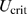 given by the log of reproductive capacity 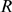 divided by the probability 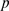 that a random mutation is deleterious. Thus, a reasonable change in mutational robustness (say, 10–30%) will result only in a minor change to 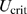. For neutral networks composed of subunits divided by barriers, we argued that barriers will in practice either be negligible or unsurmountable. In either case, a theory describing only a single neutral network is sufficient to explain how robustness affects lethal mutagenesis. We determined whether and to what extent robustness could evolve while mutagenesis was ongoing using a stochastic branching-process model of lethal mutagenesis. We found that when the initial population was small and mutation rates were high enough to be able to cause extinction, but not so high that extinction was assured, the initial neutrality of a population could affect the probability of extinction. When mutation rates were more extreme, the neutral network small, or the initial population size large, initial neutrality had little effect on the probability of extinction.

In our model of replicating RNA sequences, we found that the critical mutation rate 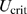 increased with increasing neutral-network size 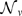. The larger the neutral network, the larger 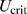. This result follows immediately from the relationship between 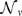 and 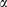. The larger 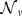, the smaller 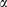 for the same 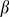. Thus, larger neutral networks are in general composed of more robust sequences that can withstand a higher mutation rate. Yet the relationship between 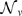 and 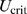 was rather weak. Increasing the neutral network size by over 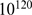-fold (from 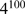 to 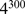) changed 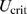 by less than a factor of 3 (Figure 6).

We found that the stochastic model behaved nearly deterministically when the initial population size was 100,000, which is not a large population for viruses. This result assumed a completely homogeneous initial population. If the initial population were heterogeneous, we would likely see nearly deterministic behavior at even lower initial population sizes. At high heterogeneity, the population might contain a single individual with high neutrality. This individual would have a low extinction probability unless 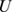 was close to 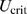. The extinction probability of the entire population would then be dominated by the extinction probability of this one individual, since the extinction probability of the entire population can only be as high as the extinction probability of any one of its members.

What are reasonable values for the fraction of deleterious mutations 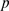
? Estimates for the fraction of lethal mutations for various viruses (VSV, poliovirus, bacteriophages) range from between 20% to 40% [18], [36], [37]. For the same viruses, between 30% and 60% of random mutations are deleterious but non-lethal [36], [37], and there seems to be a tendency for those viruses that have a higher fraction of lethal mutations to have fewer non-lethal deleterious mutations. Together, approximately 70% to 80% of random mutations are deleterious. These measurements do not provide, however, an estimate of 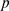 for a robust and a non-robust strain of the same virus. While such estimates are not available for entire virus genomes, several exist for individual proteins. Neutralities of less-robust variants of a protein tend to be 15% to 50% lower than neutralities of more-robust variants of the same protein [38]–[40]. If we accept an increase in robustness by a factor of two as a worst case scenario for a real-world virus, then likewise the critical mutation rate will at most double (Figure 1).

Yet mutational robustness can only increase to the extent to which it is not already present. Theory predicts that populations evolve robustness if the product of mutation rate and population size exceeds one, and that the level of robustness achieved is largely independent of the actual mutation rate [27]–[29]. For RNA viruses, whose mutation rates alone are on the order of one per genome and generation [41], we would therefore expect that their wild types have already evolved most of the robustness their genome architectures are capable of. Artificial mutagenesis should therefore not result in major additional gains in robustness for these viruses.

The reproductive capacity 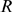 is difficult to relate to data, because it depends not only on the virus burst size but also on the number of offspring particles that go on to establish a successful infection. Burst sizes range from values in the double digits (e.g., 76 for bacteriophage 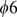 [42]) to many thousand (e.g., up to 10,000 for poliovirus [43]). Which percentage of these offspring viruses die before infecting a cell *in vivo* is unclear. More importantly, 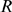 interacts with the neutral-network size to determine extinction probabilities in our stochastic models. Since we know of no precise and accurate estimates for the neutral-network size, a precise and accurate value for 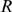 would not make the final results more meaningful. At any rate, the log-linear relationship between 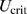 and 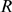 (Equation (24)) means that the change in 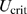 due to the evolution of robustness is not highly sensitive to the exact value of 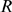.

The sequence lengths of 40 and 400 used in the stochastic models are short in comparison to the genomes of RNA viruses, which are about 10,000 base pairs long. Since the relationship between 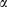 and 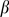 remains similar for sequences up to lengths of 10,000 (Figure S1), we expect that our analytical branching-process model gives reasonable results even when extrapolated to sequences of realistic lengths.

For our model of replicating RNA sequences under mutagenesis, we found that the critical mutation rate 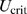 in the analytic model was slightly higher than the one in the simulations. This observation suggests that our estimates of neutral-network size 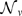 are too large. We would have overestimated 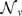 if the neutral networks for the RNA shapes chosen have multiple components, which has been observed for many RNA secondary-structure neutral networks [44]. In this case, 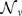 should be the size of the component, rather than the size of the entire neutral network. Alternatively, the difference in 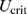 may be the result of Equation (19) not exactly matching the true fitness landscape.

The bulk of our results implies that the evolution of mutational robustness during lethal mutagenesis is not a serious threat to the efficacy of lethal mutagenesis. As long as lethal-mutagenesis treatment aims to increase 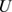 substantially beyond 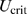 (say, to 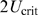 or more), the population will not be capable of compensating this increase in mutation rate by evolving a commensurate increase in robustness. This implication is consistent with the report that lymphocytic choriomeningitis virus (LCMV) passaged with a sub-lethal dose of 20 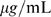 5-flourouracil (5-FU) went extinct without exception when a lethal dose of 100 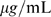 5-FU was later used [14].

Additionally, our results are not a contradiction to the report that a mutationally robust strain of vesicular stomatitis virus (VSV) prevailed in competition against a strain that was more fit in the absence of a mutagen when 5-FU doses were 20, 40, 60, and 80 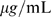 [25]. When two strains are in direct competition, relatively minor differences in robustness can favor the more robust strain over the less robust one at sub-lethal concentrations of mutagen [26], [45]. Yet both strains would likely go extinct at higher doses of mutagen.

While our models do show that the initial neutrality of a population can affect its probability of extinction, this relationship may be overshadowed in practice. For example, the models neglect the effect of defective interfering particles, which may contribute to extinction by lethal mutagenesis [13]. The defense systems of host cells or the abundance and distribution of susceptible cells could also be more important than initial population neutrality. Finally, we have not addressed the potential for resistance to the mutagen, observed in some experimental systems [23], [24].

This work has provided quantitative support for the statement that the evolution of mutational robustness will have only a minor effect on lethal mutagenesis. In an extreme case, half of all non-beneficial mutations could evolve to become neutral. In this case, doubling the mutation rate will be sufficient to cause extinction (Figure 1). For less extreme cases of robustness, less extreme increases in mutation rates would suffice. If entropic barriers to higher levels of robustness are substantial, increasing mutation rates to critical levels will not make the epochal evolution of this greater robustness appreciably more likely. If the entropic barriers are small and virus population sizes are appreciable, we generally need to treat the population as if it consisted of viruses with the mutation-selection–equilibrium level of robustness. So while natural selection may increase the sequence neutrality of viruses during lethal mutagenesis, by itself, this effect is unlikely to affect the course of treatment. The analysis of the potential effects of increased sequence neutrality combined with the evolution of higher-fidelity polymerases and other compensatory mutations remains a topic for future work.

## Methods

### Numerical evaluation of analytic results

We evaluated the convergence times given by Equation (8), numerically derived 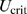 from Equation (5), and implemented a bisection root finding algorithm to solve Equations (19) and (20) for 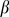, given all other parameters, using the Sage [46] computing environment. Specific components of Sage used included the multiple-precision library MPFR [47], SciPy [48], and the computer algebra system Maxima [49]. The scripts used are included in Dataset S1.

We obtained the fixed point 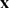 in Eq. 12 by iterating the p.g.f.s until the total difference between the input vector and the resulting vector was less than 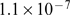. Component 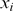 of 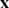 gives the extinction probability of a population that begins with a single sequence of type 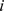. To calculate the extinction probabilities of populations of size 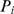 where 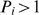, we assumed independence of the extinction of each lineage in the initial population (consistently with the branching process) and used the probability that all of the lineages went extinct, 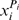.

### Simulations on RNA secondary-structure networks

Sequences that folded into a target shape were considered neutral, and all others were considered inviable. The neutrality of a sequence was the fraction of neighbors at a Hamming distance of one that also had the target phenotype. The RNAfold function in the Vienna package [50] version 1.7 was used for the folding. Unpaired bases were allowed to participate in at most one dangling end (the default option -d1). The size of the neutral network was determined by randomly sampling the sequence space and seeing what proportion was neutral, and then multiplying this proportion by the size of the sequence space. We chose target shapes that were relatively common and limited the sequence length to 40. This limit reduced the number of random sequences that needed to be sampled to estimate the neutral-network size without introducing any obvious biases in the results. We used the following targets:

1. ((((....))))............................
2. .(((..........(((((.....))))))))........

Here, positions that form base pairs are indicated with matching parentheses, and unpaired positions are indicated with dots. For the first target, which was used to generate the results in Figure 5A, we sampled two hundred million sequences and found 88,840 to be neutral. Therefore, 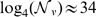. For the second target, which was used to generate the results in Figure S2A, we sampled one hundred billion sequences and found 19,782 to be neutral. Therefore, 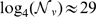.

The extinction probability of a sequence was determined by simulation of a branching process on the RNA secondary-structure neutral network. Simulations began with a single neutral sequence. These sequences were selected from the sample of sequences used to estimate the size of the neutral network so as to get the full range of initial neutralities. At each iteration, each sequence in the population had a Poisson distributed number of offspring. Each letter of the sequence changed to any of the other three possible letters with a probability equal to the genomic mutation rate divided by the sequence length. Mutation rates ranged from zero to fifteen. Each sequence was tested to see if it folded into the target, and sequences that did not were removed. Simulation was continued until the population size reached zero or 10,000. Simulations were replicated 100 times for each of 500 initial sequences and the extinction probability was the number of simulations in which extinction occurred divided by the total number of simulations. A local polynomial fitting function (the loess function in R [51]) was used to produce smooth curves from the extinction probability data. In Figure 5A, the maximum mutation rate used in simulation runs was 15. The extinction probability for larger mutation rates is an extrapolation of the observed pattern. We have no reason to expect that this extrapolation is incorrect.

The code written for these analyses is in Dataset S1.

## Supporting Information

Figure S1Negative correlation between neutrality and epistasis. Equations (19) and (20) predict that as the parameter α of a sequence increases, the epistasis parameter β decreases. The panels are labeled with the sequence lengths of 40, 5000, and 10000. For each sequence length, the lines, from highest to lowest, are numerical solutions where log_4_(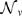) was set to approximately one third, two thirds, and five sixths of the sequence length. This gave neutral-network sizes equivalent to those from fitness landscapes in which approximately one third of sites are neutral, two thirds of sites are neutral, and the same proportion of sites are neutral as for the sequences in the RNA simulations in Figure 5.(0.02 MB EPS)

Figure S2Extinction probability as a function of initial neutrality and deleterious genomic mutation rate. Panel A displays results from simulations where sequence neutrality was determined by RNA folding. Panel B displays results from a branching process model derived from the correlation between sequence neutrality and epistasis. Only in a band of intermediate mutation rates does the extinction probability depend on initial neutrality 1-*p*. Parameters: sequence length *l* = 40, neutral-network size 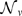
≈4^29^, reproductive capacity *R* = 50, initial population size = 1 sequence.(0.26 MB EPS)

Dataset S1 Raw data and computer code necessary to reproduce all results reported in this paper.
(0.46 MB ZIP)

Text S1 Supplementary text.
(0.06 MB PDF)
