# Supplementary material for: Does Mutational Robustness Inhibit Extinction by Lethal Mutagenesis in Viral Populations?
Source: PLoS Comput Biol. 2010 Jun 10;6(6):e1000811. doi: 10.1371/journal.pcbi.1000811 (PMC2883602; doi:10.1371/journal.pcbi.1000811)
Supplement: Text S1 — Supplementary text. (0.06 MB PDF) [file pcbi.1000811.s004.pdf]

This is an alternative derivation of the probability generating function used to calculate extinction probabilities in the analytic model.

As explained in the main text, the probability that any one offspring of a parent of type i is of type r is

$$M_{ir} = \sum_{n=0}^{l} m_n w_i(n) d_r(n), \tag{1}$$

where m_n is the probability of having n mutations, $w_i(n)$ is the probability that an offspring of a type i sequence with n mutations is viable, and $d_r(n) = 1$ if $1 - w_i(n+1)/w_i(n)$ is in $(p_{r-1}, p_r]$ and $d_r(n) = 0$ otherwise.

The probability, then, of a parent producing k_r offspring of type r, given that the parent is of type i and that the parent produces a total of K offspring, is binomially distributed as $P(k_r|i,K) = \binom{K}{k_r}(M_{ir})^{k_r}(1-M_{ir})^{K-k_r}$. Likewise, the probability of a parent producing any combination of types of offspring given that the parent is of type i and produces a total of K offspring is multinomially distributed as

$$P(k_1,\ldots,k_B|i,K) = \frac{K!}{(K-\sum_r k_r)!\prod_r k_r!}\left(1-\sum_{r=1}^{B} M_{ir}\right)^{K-\sum_r k_r}\prod_{r=1}^{B}(M_{ir})^{k_r}. \tag{2}$$

To fully specify Equation (2), we assume that the distribution of mutations m_n in Equation (1) is Poisson with mean U, i.e. $m_n = e^{-U}U^n/n!$. We also assume that number of offspring K is a Poisson random variable with mean R. To get an offspring distribution that is not conditional on K, we sum Equation (2) over all possible values of K and weight each term with the probability of that K:

$$\begin{aligned} P(k_1,\ldots,k_B|i) &= \sum_{K=0}^{\infty} P(k_1,\ldots,k_B|i,K)e^{-R}\frac{R^K}{K!} \\ &= e^{-R}\prod_{r=1}^{B}\left(\frac{(RM_{ir})^{k_r}}{k_r!}\right)\sum_{K=\sum_r k_r}^{\infty}\frac{R^{K-\sum_r k_r}\left(1-\sum_r M_{ir}\right)^{K-\sum_r k_r}}{(K-\sum_r k_r)!} \\ &= \prod_{r=1}^{B}\left(e^{-RM_{ir}}\frac{(RM_{ir})^{k_r}}{k_r!}\right). \end{aligned} \tag{3}$$

So we can express the offspring distribution as a product of Poisson random variables with means RM_{ir}.

Now we calculate the p.g.f. that corresponds to the offspring distribution. A multivariate p.g.f is defined as

$$f_i(\mathbf{z}) = \sum_{k_1,\ldots,k_B} P(k_1,\ldots,k_B|i) z_1^{k_1}\cdots z_B^{k_B}. \tag{4}$$

Inserting Equation (3) into Equation (4) and simplifying gives:

$$\begin{aligned} f_i(\mathbf{z}) &= \sum_{k_1,\ldots,k_B}\prod_{r=1}^{B}\left(e^{-RM_{ir}}\frac{(RM_{ir}z_r)^{k_r}}{k_r!}\right) \\ &= \exp\left[\sum_{r=1}^{B} RM_{ir}(z_r-1)\right]. \end{aligned} \tag{5}$$
